# Persistent elevations of alkaline phosphatase as an early indicator of GM1 gangliosidosis

**DOI:** 10.1016/j.ymgmr.2025.101191

**Published:** 2025-01-20

**Authors:** Iskren Menkovic, Monika Williams, Neelam Makhijani, Ruhan Wei, Sarah P. Young, Areeg El-Gharbawy, Ashlee R. Stiles

**Affiliations:** a Biochemical Genetics Laboratory, Duke University Health System, Durham, NC, USA; b Division of Medical Genetics, Department of Pediatrics, Duke University Medical Center, Durham, NC, USA; c Department of Pathology, Duke University Medical Center, Durham, NC, USA; d Duke University Health System Clinical Laboratories, Durham, NC, USA

**Keywords:** GM1 gangliosidosis, Alkaline phosphatase, Keratan sulfate, Skeletal survey, Glycosaminoglycan

## Abstract

*GLB1*-related disorders are autosomal recessive lysosomal diseases caused by enzymatic deficiency of β-galactosidase. Enzymatic deficiency of β-galactosidase may lead to one of two phenotypes, GM1 gangliosidosis or mucopolysaccharidosis IVB (MPS IVB). GM1 gangliosidosis is a neurodegenerative disorder with variable skeletal disease and involvement of other systems. The age of onset correlates with the extent of neurological involvement and established genotype/phenotype correlations. Mucopolysaccharidosis IVB is characterized by a skeletal dysplasia without neurological involvement. Diagnostic work-up for *GLB1-*related disorders includes enzyme analysis, biomarker analysis, molecular testing, and laboratory imaging studies.

We report a patient who presented with persistent elevations of alkaline phosphatase (ALP) and subtle dysmorphic facial features. An initial skeletal survey at birth was unrevealing; however, a repeat at 3 months of age was abnormal with anterior beaking of the lumbar vertebrae and hemivertebrae of the lower cervical spine. Urinary glycosaminoglycan (GAG) analysis revealed a marked elevation of keratan sulfate (KS). Clinical exome sequencing revealed pathogenic heterozygous variants in *GLB1*, consistent with *GLB1*-related GM1 gangliosidosis.

Our case demonstrates that persistent elevations of ALP may be an early indicator for GM1 gangliosidosis in an infant with progressive multisystem disease, indicating the need for early genetic consultation. This case also highlights the utility of repeat skeletal surveys with abnormalities detected at 3 months of age.

## 1 Introduction

GM1 gangliosidosis (MIM: 230500, 230600, 230650) is a lysosomal disease caused by biallelic pathogenic variants in *GLB1* which encodes β-galactosidase (EC 3.2.1.23) [1,2]. β-galactosidase catalyzes the removal of terminal β-linked galactose from glycolipids and oligosaccharides, resulting in an accumulation of GM1 ganglioside and disruption of normal brain metabolism and homeostasis [3]. The resulting phenotypes range from severe psychomotor delays, organomegaly, skeletal abnormalities, and death in early childhood as observed in GM1 gangliosidosis type 1 (infantile form) to a late-onset form of limb-girdle and neuromuscular weakness, ataxia, and skeletal abnormalities as observed in GM1 gangliosidosis type 3 (adult form) [[4], [5], [6]]. GM1 gangliosidosis type 2 can be separated into late-infantile and juvenile forms. In the late-infantile form, the patient may achieve developmental milestones followed by psychomotor regression beginning around 12 months of age with death occurring in the 2nd decade of life. In the juvenile form of the condition, development may be normal until 3 to 5 years of age [[4], [5], [6]]. There are currently no approved treatments for GM1 gangliosidosis [7]. Laboratory anomalies may include persistently elevated alkaline phosphatase (ALP) and abnormal aspartate aminotransferase with normal/elevated alanine transaminase. Keratan sulfate (KS) is typically elevated, and urine oligosaccharide analysis may show elevated galactosylated free oligosaccharides [6,8,9].

Herein, we describe the clinical presentation of a patient with GM1 gangliosidosis type 1 diagnosed at an early age with diagnostic clues that included persistent alkaline phosphatase noted from the third week of life and the development of coarse facial features and an abnormal skeletal survey, identified at an early age of three months.

## 2 Methods

The clinical team reviewed the patient's medical record and clinical parameters.

Urinary GAG analysis was performed using liquid chromatography-tandem mass spectrometry by Duke University Health System's (DUHS) CLIA/CAP-certified Biochemical Genetics Laboratory according to standard operating procedures. Chondroitin sulfate (CS), dermatan sulfate (DS), and heparan sulfate (HS) were analyzed as methylated dimers following hydrolysis of GAGs using methanolic hydrochloride, according to a published method [10]. Analysis of KS dimers was performed following enzymatic digestion, as published [9,11,12]. Creatinine analysis was performed by the alkaline picrate (Jaffe reaction) method.

Serum total ALP analysis was performed by the DUHS CLIA/CAP-certified Central Automated and Clinical Pediatrics Laboratories. ALP activity was measured using the Beckman Coulter (Chaska, MN) UniCel® DxC800 System. ALP converts p-nitrophenylphosphate to yellow-colored p-nitrophenol in an alkaline solution. p-Nitrophenol is measured spectrophotometrically, and the change in absorbance is directly proportional to ALP activity. ALP isoenzyme analysis was conducted using the Sebia HYDRASYS 2 (Norcross, GA) instrument. The isoenzymes were electrophoresed through an alkaline-buffered agarose gel, separated based on charge differences, and quantified by a densitometer. Serum ALP isoenzyme analysis was performed by Mayo Clinic Laboratories.

## 3 Case report

### 3.1 Clinical presentation

Prenatal ultrasound imaging revealed several cardiac abnormalities including a moderately dilated right atrium and ventricle, tricuspid valve regurgitation, and a tortuous ductal arch with mildly elevated velocities and right to left shunting. Fetal movement and amniotic fluid volume were normal. The patient was born at full term by spontaneous vaginal delivery. Apgar scores were recorded as 8 and 9 at 1 and 5 min, respectively. Upon delivery, a physical exam revealed a heart murmur; however, no dysmorphic features were described. A transthoracic echocardiogram detected several congenital cardiac anomalies, including ventricular septal defect, patent foramen ovale, and small patent ductus arteriosus. A head ultrasound revealed a grade I intraventricular hemorrhage which was visualized by brain magnetic resonance imaging; however, no other brain pathology was identified. The patient developed respiratory insufficiency soon after birth, requiring supplemental oxygen (0.5–2 L oxygen via nasal cannula) and intermittent periorbital edema of unknown etiology requiring intermittent doses of Lasix (furosemide). Chest radiograph showed a deviated trachea, suggestive of a mediastinal mass, prompting the patient's transfer to DUHS for further evaluation and care.

Upon arrival at DUHS, a chest ultrasound and a computed tomography scan did not detect the mediastinal mass. A repeat chest x-ray confirmed a deviated trachea and cardiac enlargement. Non-sedated auditory brainstem response revealed mild to moderate hearing loss in the right ear and severe hearing loss in the left ear for frequencies 500–4000 Hz.

Due to elevated ALP, endocrinology was consulted. Transient hyperphosphatasemia in infancy and early childhood was initially suggested as the working diagnosis; however, further work-up excluded the diagnosis.

Medical genetics consultation soon after birth revealed that the family history was non-contributory. A marked elevation of plasma ALP was first documented at 16 days of age, and ALP remained persistently elevated over the next 3 weeks (Table 1). ALP fractionation showed a predominant elevation of the bone isoenzyme (73.1 %; Table 2). Other bone mineral markers were essentially within reference limits (Table 1). Given the elevated ALP, a next-generation gene sequencing panel for Mabry disease, which included: *PIGV, PIGO, PIGL, PIGY, PGAP2, PGAP3,* and *PIGW,* was sent and was negative.

**Table 1:** Bone-related markers reveal elevated plasma alkaline phosphatase.

Age Collected (Days)	16	22	39	34	52	55
Alkaline Phosphatase (U/L) Reference range: 110–365 U/L	1199	1043	1029	1124	1241	1388
25 Hydroxy-Vitamin D (ng/mL) Reference range: 30–100 ng/mL	–	57	–	–	47	–
Phosphorus (mg/dL) Reference range: 4.2–8.2 mg/dL	7.1	5.7	–	6.2	–	6.9
Calcium (mg/dL) Reference range: 7.0–11.0 mg/dL	10	9.9	–	9.8	10.3	10.2
Parathyroid Hormone (pg/mL) Reference range: 14–72 pg/mL	–	–	–	–	–	73

**Table 2:** Alkaline phosphatase isoenzyme analysis collected on day of life 55.

Component	Concentration (U/L)	Percentage (%)
Total Alkaline Phosphatase Reference range: 122–469	1639	100
Liver Isoenzyme 1 Reference range: 7.0–112.7	0	0
Liver Isoenzyme 2 Reference range: 3.0–41.5	83.6	5.1
Bone Isoenzyme Reference range: 43.5–208.1	1198.1	73.1
Intestine Isoenzyme Reference range: 0.0–37.7	357.3	21.8

**Fig. 1:**
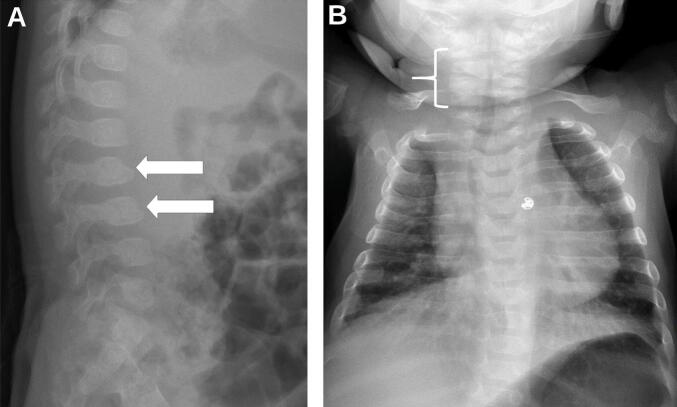
Skeletal Survey: (A) Lateral thoracic and (B) frontal thoracic.

Follow-up visit with medical genetics at almost 3 months of age showed persistent central hypotonia and head lag and hypertonia in the extremities. The patient had bilateral metatarsus adductus and externally rotated hips, as well as coarsening of the facial features prompting a repeat skeletal survey. There was no evidence of acute or healing fracture; however, anterior beaking of a few lumbar vertebral bodies (Fig. 1a) and hemivertebrae of the lower cervical spine (Fig. 1b) were noted with normal disc space heights, suggestive of a mucopolysaccharidosis (MPS). No evidence of dysostosis multiplex was noted at this time, as depicted in Fig. 1. Urinary GAG analysis was ordered and revealed an isolated elevation of KS. Other GAGs (CS, DS, and HS) were within reference limits (Table 3). Clinical exome sequencing analysis identified two heterozygous pathogenic variants in *GLB1*: c.245 + 1 G > A (p.?; ClinVar accession VCV000417873.6) and c.202C > T (p.Arg68Trp; ClinVar accession VCV000000944.7), confirming the diagnosis of autosomal recessive GLB1-related GM1 gangliosidosis type 1.

**Table 3:** Urinary glycosaminoglycan results.

Glycosaminoglycan Species	Result (g/mol creatinine)	Reference Range (g/mol creatinine)
Chondroitin Sulfate (CS)	5.9	≤ 10.7
Dermatan Sulfate (DS)	2.4	≤ 8.3
Heparan Sulfate (HS)	21.3	≤ 55.6
Keratan Sulfate (KS)	5.3 (High)	≤ 1.6

## 4 Discussion

We describe the early clinical presentation and diagnostic clues that resulted in the diagnosis of infantile-onset GM1 gangliosidosis (type 1) at 3 months of age. Our patient presented with prenatal cardiac anomalies, followed by a progressive multisystem disease. Markedly elevated ALP was documented at 16 days of life. While this may be concerning and raise suspicions for a broad differential diagnosis, including endocrine and inherited bone-related disorders given the high ALP [13,14], all other biochemical markers associated with bone metabolism (parathyroid hormone, vitamin D, calcium, phosphorus) yielded normal findings. Isolated elevations in ALP have been described in children with transient hyperphosphatasemia [15], a benign condition characterized by markedly elevated ALP levels (up to 20× the pediatric upper reference limit) in the absence of bone or liver disease [16]. Given the progressive multisystem clinical nature of the disease with coarsening facial features, and abnormal spine findings, lysosomal diseases associated with bone abnormalities were considered in the differential.

Whereas the initial skeletal survey performed soon after birth was unrevealing, vertebral anomalies were detected 3 months later and were suggestive of a MPS disorder. The identification of an isolated, elevation of KS, without elevation of CS, using sensitive and specific LC-MS/MS methods for analyzing GAGs, narrowed the diagnosis to a GLB1-related disorder (MPS IVB, MIM: 253010 or GM1 gangliosidosis). A diagnosis of MPS IVA (*N*-acetylgalactosamine sulfate sulfatase deficiency, MIM: 253000) was less likely, as this disorder is typically characterized by elevations of chondroitin-6-sulfate in addition to KS [17].

Elevated ALP has previously been reported as an early finding in a small number of cases infantile GM1 gangliosidosis, including two siblings who both presented at birth and a 4-month old who presented with edema [18,19]. ALP has also been shown to be significantly higher in infantile GM1 gangliosidosis compared with late-infantile and juvenile cases [20].

Although MPS IVB and GM1gangliosidosis share overlapping clinical features, the skeletal findings were more suggestive of the GM1 gangliosidosis, which was confirmed molecularly with two pathogenic variants detected in *GLB1*. The c.245 + 1 G > A (p.?) variant leads to a premature termination codon starting at position c.266 in exon 3, severely affecting the resulting transcript of *GLB1* which contains 16 exons [21,22]; and while the c.202C > T (p.Arg68Trp) variant does not appear to interfere with gene transcription or translation, *in-silico* prediction algorithms suggests that this variant may affect the precursor processing and the capacity of GLB1 to form the active tetramer complex and/or the catalytic site of the enzyme [23] leading to disease.

As there are currently no FDA-approved treatments for GM1 gangliosidosis type 1, and the patient was diagnosed early (before 4 months of age); attempts were made to enroll our patient in an open active gene therapy clinical trial for GM1 gangliosidosis. Unfortunately, due to a lack of funding and the current trial policies, none of the clinical trials remained open for recruitment at the time of the patient's diagnosis. We counseled the family about the genetic disorder, recurrence risk, and long-term morbidity and mortality concerns, and discussed the disease process and the need for symptomatic management of the condition; a palliative care team was also consulted. Additionally, in an attempt to delay the disease process, given the early diagnosis, Miglustat, a glucosylceramide synthase inhibitor was discussed as an off label therapeutic intervention. Studies performed on GM1 gangliosidosis mice treated with Miglustat revealed significantly decreased neuroinflammation and functional improvement [24] and GM1 gangliosidosis types 2 and 3 patients report clinical improvement with treatment [25]. Due to parental concerns that Miglustat may not change the outcome, the parents declined treatment. Upon follow-up over 6 months, the patient continued to show signs of progression of the natural history of the disease. This included worsening cardiac findings that were not amenable to surgery, severe truncal hypotonia and head lag, distal hypertonia, increased irritability, discomfort and global developmental delay. The patient was fed via gastrostomy tube due to poor feeding and aspiration risk and continued to decline with less interaction, increased seizure activity and intermittent apneic episodes. Inability to tolerate feeds and increased oxygen requirements led to hospitalization at 13 months of age with respiratory failure managed with BiPAP and later transitioned to high flow nasal canula. Renal failure developed with progressive epileptic encephalopathy associated with decreased responsiveness. Ultimately, care was transitioned to comfort care, maximizing pain control, until she passed away at 15 months of age.

## 5 Conclusion

This case raises awareness of early presentations of lysosomal diseases and highlights the significance of considering the diagnosis of GM1 gangliosidosis in the differential diagnosis of elevated ALP in the presence of multisystem disease. The repeated close clinical evaluation of the infant by a geneticist detected the coarsening features over time, as well as the abnormal bone findings indicating the gradual progression which is characteristic of storage disorders. Finally, this case highlights the continued urgent need to develop new therapies for GM1 gangliosidosis, as an early diagnosis may be achieved by clinical suspicion and current molecular and enzyme testing strategies. A multidisciplinary approach, that included consultation and follow-up with medical genetics early-on in the course of the disease, were critical in making a timely diagnosis of this rare disorder with multisystem involvement. Despite an early diagnosis, the lack of a treatment led to progression of the natural history of the disease.

## CRediT authorship contribution statement

**Iskren Menkovic:** Writing – original draft, Methodology, Investigation, Formal analysis, Data curation, Conceptualization. **Monika Williams:** Writing – original draft, Investigation. **Neelam Makhijani:** Investigation, Formal analysis. **Ruhan Wei:** Methodology, Data curation. **Sarah P. Young:** Writing – review & editing. **Areeg El-Gharbawy:** Writing – review & editing, Visualization, Supervision. **Ashlee R. Stiles:** Writing – review & editing, Writing – original draft, Visualization, Supervision, Methodology, Formal analysis, Conceptualization.

## Declaration of competing interest

None.

## Data Availability

The authors do not have permission to share data.
